# Utilizing metabolomics and network analysis to explore the effects of artificial production methods on the chemical composition and activity of agarwood

**DOI:** 10.3389/fphar.2024.1357381

**Published:** 2024-05-07

**Authors:** Wencheng Hou, Jian Feng, Yuanyuan Sun, Xiqin Chen, Yangyang Liu, Jianhe Wei

**Affiliations:** ^1^ Hainan Provincial Key Laboratory of Resources Conservation and Development of Southern Medicine and Key Laboratory of State Administration of Traditional Chinese Medicine for Agarwood Sustainable Utilization, Hainan Branch of the Institute of Medicinal Plant Development, Chinese Academy of Medical Sciences and Peking Union Medical College, Haikou, China; ^2^ Key Laboratory of Bioactive Substances and Resources Utilization of Chinese Herbal Medicine, Ministry of Education and National Engineering Laboratory for Breeding of Endangered Medicinal Materials, Institute of Medicinal Plant Development, Chinese Academy of Medical Sciences and Peking Union Medical College, Beijing, China

**Keywords:** agarwood, agarwood-inducing technique, volatile metabolite, metabolomics, network analysis

## Abstract

**Introduction:** Agarwood is a traditional aromatic southern medicine. It has a long history of being used in traditional Chinese aromatherapy to treat insomnia, anxiety and depression. Due to the scarcity of wild resources, people have planted trees successfully and begun to explore various agarwood-inducing techniques. This study comparative analysis of volatile metabolites in agarwood produced by various inducing techniques and its potential sleep-promoting, anti-anxiety and anti-depressant network pharmacological activities.

**Methods:** A total of 23 batches of two types of agarwood were collected, one of which was produced by artificial techniques, including 6 batches of TongTi (TT) agarwood produced by “Agar-Wit” and 6 batches of HuoLao (HL) agarwood produced by “burning, chisel and drilling”, while the other was collected from the wild, including 6 batches of BanTou (BT) agarwood with trunks broken due to natural or man-made factors and 5 batches of ChongLou (CL) agarwood with trunks damaged by moth worms. The study employed metabolomics combined with network analysis to compare the differences in volatile metabolites of agarwood produced by four commonly used inducing techniques, and explored their potential roles and possible action targets in promoting sleep, reducing anxiety, and alleviating depression.

**Results:** A total of 147 volatile metabolites were detected in agarwood samples, mainly including small aromatic hydrocarbons, sesquiterpenes and 2-(2-phenylethyl) chromone and their pyrolysis products. The results showed composition of metabolites was minimally influenced by the agarwood induction method. However, their concentrations exhibited significant variations, with 17 metabolites showing major differences. The two most distinct metabolites were 6-methoxy-2-(2-phenylethyl) chromone and 6,7-dimethoxy-2-(2-phenylethyl) chromone. Among the volatile metabolites, 142 showed promising potential in treating insomnia, anxiety, and depression, implicating various biological and signaling pathways, predominantly ALB and TNF targets. The top three active metabolites identified were 2-(2-phenylethyl) chromone, 1,5-diphenylpent-1-en-3-one, and 6-methoxy-2-[2-(4'-methoxyphenyl) ethyl] chromone, with their relative content in the four types of agarwood being TT>HL>CL>BT.

**Conclusion:** The differences in the content of 2-(2-phenylethyl) chromones suggest that they may be responsible for the varying therapeutic activities observed in different types of agarwood aromatherapy. This study offers theoretical support for the selection of agarwood in aromatherapy practices.

## 1 Introduction

Neurological diseases such as insomnia, anxiety, and depression are major problems affecting human health in the 21st century that cannot be ignored (Bourgin et al., 2020; Ballesio et al., 2022). Traditional Chinese medicine aromatherapy is a kind of characteristic therapy in which aromatic medicinal materials are heated to promote the release of the volatile active metabolites of the medicinal materials, achieving a curative effect through the patient’s breathing and inhalation (Simsek and Alpar, 2022; You et al., 2022). Agarwood has a long history of application in aromatherapy (Ma et al., 2021), their aromatherapeutic effects are sedative and soothing, sleep-promoting, anti-anxiety, anti-depression and other neurological activities (Rothan et al., 2014; Wang et al., 2022). It is resinous wood obtained from wounded trees of *Aquilaria* and *Gyrinops* spp., which only produced when the plant is damaged (Li, 2004; Lu, 2016).

Different damage can produce different agarwood, which were mainly divided into natural damage and artificial induction according to the source of damage. Natural damage mainly consists of being moth-eaten and stem breakage, which can form ChongLou (CL) and BanTou (BT), respectively (Gu et al., 2017; Azren et al., 2019). The most primitive form of medicinal agarwood, which has low efficiency and low yield, cannot meet the needs of modern medicinal agarwood use (Wang et al., 2020). Thus, agarwood produced by artificial induction techniques has become the main supply form of modern medicinal raw materials. These techniques are represented by agarwood-whole inducing technology (Agar-Wit) (Liu et al., 2013), burning-chisel-drilling (Wei et al., 2009) and inoculation with agarwood-producing fungi (Ma et al., 2021). The most commonly used are the first two methods, which produce HuoLao (HL) and TongTi (TT) agarwood, respectively.

The agarwood produced by different methods has different characters and tastes, which means that there may be differences in biological activity, this has been confirmed by research reports (Wang et al., 2017). The main active metabolites are sesquiterpenes and small molecule aromatic hydrocarbons according to the literature (Forrester et al., 2014; Farrar and Farrar, 2020), however, there is currently no comparative study on the differences in the volatile metabolites of agarwood produced by different methods and the aromatherapy activity of the 2-(2-phenethyl) chromones they contain, which leads to confusion in the use of different agarwood types and large differences in efficacy. Therefore, the comparative study of the effects of agarwood formation methods on the volatile metabolites of agarwood has important guiding significance for the cultivation, production and application of agarwood.

In recent years, metabolomics has been developed and applied to a variety of botanical drug origins (Miao et al., 2022), processing methods (Zhang et al., 2022a) and species (Zhang et al., 2022b), caused their systematic large-scale profiling, they can characterize the chemical compositions of different samples through targeting or non-targeting detection of metabolites. While the combined method of both metabolomics and network analysis enables effective screening of medicinal variety or medicinal part. Zhu et al. (2024) employed a disease model-oriented network pharmacology approach coupled with targeted metabolomics to evaluate the medicinal properties of nine *Rhodiola* species. Their findings demonstrated that *Rhodiola kirilowii* (Regel) Maxim. Emerged as the most promising medicinal option. Wang et al. (2023) utilized a combination of “GC-MS fingerprinting - network pharmacology - chemometric pattern recognition” to demonstrate that *Atractylodes lancea* (Thunb.) DC. cannot substitute for *Atractylodes chinensis* in medicinal use. However, cultivated *A. chinensis* can replace wild *A. chinensis* for medicinal purposes.

This study collected a total of 11 batches of natural agarwood and 12 batches of artificially induced agarwood, their volatile metabolites were determined and compared, and the potential sleep-promoting, anti-anxiety and anti-depressant activities were studied by a network analysis method. The results showed that different agarwood-inducing techniques have little effect on the composition of the volatile metabolites of agarwood they produce but a significant impact on the relative content of each metabolite, 2-(2-phenylethyl) chromones and their cracking products also play an important role in the sleep-promoting, anti-anxiety and anti-depressant activities of agarwood.

## 2 Materials and methods

### 2.1 Agarwood samples

The sample information in this experiment is shown in Supplementary Table S1. The agarwood samples were smashed and passed through a No. 3 sieve (50 mesh) after impurity removal. All sample specimens were stored in the sample library of the Agarwood Identification Center of Hainan Branch, Institute of Medicinal Botany, Chinese Academy of Medical Sciences.

### 2.2 Chemical reagents

(+)-AroMadendrene (CAS: 489-39-4, 99.95%) was purchased from Pufei De Co., Ltd (Chengdu, China) for mass spectrometry accuracy testing. Standard compounds of n-alkanes (C7-C30) were purchased from Sigma‒Aldrich (St. Louis, MO, USA) for determining their corresponding retention indices (RIs). Acetone (CAS: 67-64-1, 99.50%) was analytical grade and purchased from Xilong Chemical Co., Ltd. For volatile metabolite extraction of agarwood samples.

### 2.3 Metabolomics study

#### 2.3.1 Sample extraction

A total of 0.100 g of agarwood powder was precisely weighed, and 10 mL of acetone was added to ultrasonically extract the sample for 30 min. The resulting solution was filtered, and 1 mL of acetone was added to rinse the residue twice. The filtrates were filtered, combined, and diluted to 10 mL. A total of 0.5 mL of each of the 23 batches of sample extracts was mixed well as a quality control (QC) sample, and acetone was used as a blank sample (CK). Before being used for GC‒MS analysis, all samples were filtered with a 0.22 μm membrane filter.

#### 2.3.2 GC‒MS analysis conditions

The GC‒MS instrument was an Agilent 7890B-5975C, and an Agilent HP-5MS chromatographic column (30 m × 0.25 mm, 0.25 μm) was used for separation. The inlet temperature was 250°C. The carrier gas was ultrahigh purity helium (99.9995%) at a flow rate of 1 mL/min. The heating program was as follows: the initial temperature was 50°C, held for 1 min, then increased to 140°C at a rate of 15°C/min, held at 140°C for 8 min, increased to 155°C at a rate of 1°C/min, held at 155°C for 8 min, ramped up to 175°C at a rate of 10°C/min, held at 175°C for 7.5 min, ramped up to 200°C at a rate of 5°C/min, held at 200°C for 9.5 min, held at a rate of 20°C/min raised to 260°C, maintained at 260°C for 5 min, splitless injection, detection range 30-550 m/z.

#### 2.3.3 Quality control sample preparation

The data quality of the metabolomic study on the effect of agarwood formation on the metabolites of agarwood was ensured by the following aspects: before sequence analysis, blank and single standard samples were injected, the cleanliness of the GC‒MS system was checked, and the system was balanced. QC samples were injected before and after sample analysis, and QC samples were injected once every 5 experimental samples during the sequence analysis. The extracts of agarwood produced by different formation methods and blank samples were randomly injected, and the RI was analyzed by injection for C_7_-C_30_ n-alkane analytical calculations in the form of mixtures.

### 2.4 Network analysis

#### 2.4.1 Metabolites and disease target selection

The InChIs of all metabolites analyzed by the National Institute of Standards and Technology (NIST) 17.0 database were converted into SMLIES, and SwissTargetPrediction was used to predict them to obtain the target of the metabolite and select the target gene with probability>0. The Online Mendelian Inheritance in Man (OMIM) and Genecards databases were used to search with the keywords “insomnia, sleep disorders, sleepiness, fatigue sedative, depression, depressive disorder, anxiety and anxiolytic” to obtain disease targets. The Venny 2.1.0 online analysis tool was used for analysis. After entering the drug target and the disease target into the software, the common drug-disease target was obtained after taking the intersection (Zou et al., 2020).

#### 2.4.2 Construction of the protein interaction network

The intersection targets analyzed in 2.4.1 were uploaded to the Search Tool for the Retrival of Interacting Genes/proteins (STRING) database (https://cn.string-db.org/) to construct a protein‒protein interaction (PPI) network, and the species was set to “*Homo sapiens*”. The PPI network was obtained, and the PPI network TSV file was imported into Cytoscape 3.9.0 software for visualization.

#### 2.4.3 Gene Ontology (GO) and kyoto encyclopedia of genes and genomes (KEGG) analysis

The cluster Profiler software package was used to analyze the common targets analyzed in 2.4.1 by GO database (http://www.geneontology.org/) and KEGG database (http://www.genome.jp/kegg/) analysis, and the top 20 entries were screened according to the number of target genes and *p*-value (*p* < 0.05). Visual analysis was performed using the “Bioinformatics” online platform.

#### 2.4.4 Molecular docking verification

The Program DataBase (PDB) file of the 3D structure of the key target protein was downloaded from the PDB database, and PyMOL Version 2.0 was used to perform operations such as dehydration and hydrogenation. The compound ligand uses RDKit to convert its corresponding SMILES into a 3D structure PDB file, and finally, MGLTOOLS version 1.5.7 converts PDB files of receptors and ligands to PDBQT format. Then, using proteins as receptors and small molecules as ligands, according to the prediction results of Proteins Plus, the active pocket with the highest DrugScore score was selected as the active site for molecular docking, and AutoDock Vina software 2.1.0 was used to predict the binding ability. The lower the binding energy is, the more stable the conformation, and the top 4 histones and metabolites with the best binding activity were selected for visual display using PyMOL Version 2.0.

### 2.5 Data processing and statistical analysis

The raw data were exported to a. CDF format file via OpenLab ChemStation (Agilent, USA), imported into MS-DIAL (NSF-JST, Japan) for peak alignment, dewrapped and exported to a metabolite list using SIMCA-P 14.1 (Umetrics, Umea, Switzerland) for principal component analysis (PCA) and Orthogonal Partial Least Squares-Discriminant Analysis (OPLS-DA). A t-test for differential metabolites was performed with SPSS 22.0 (IBM, USA), and graphs were drawn with Origin 2019b (OriginLab, USA).

## 3 Results

### 3.1 Effect of agarwood inducing method on volatile metabolite content

**FIGURE 1:**
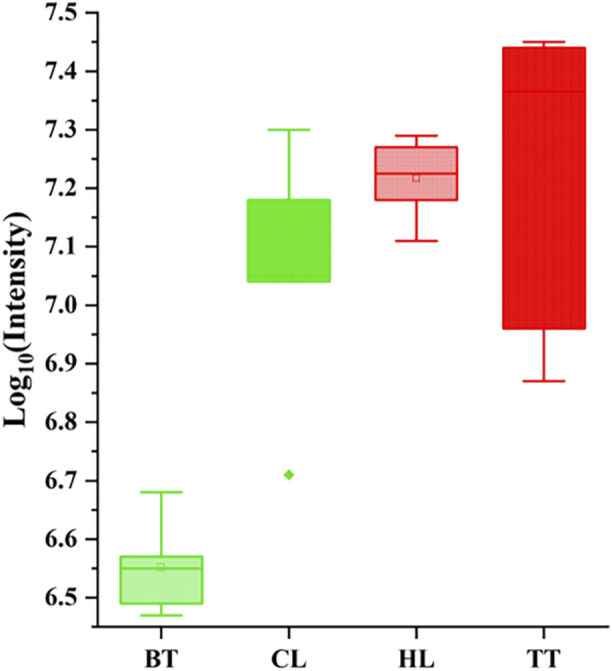
Contents of volatile metabolites in four agarwood samples.

The total ion chromatogram (TIC) reflects the sum of all ion intensities that can be generated by the sample at the detection time. When the sample is treated in the same way, its change is positively correlated with the metabolite content in the sample, so its peak area can be used to evaluate the content of the metabolite in the sample. In this study, the logarithm of the TIC total peak area of agarwood samples produced by four agarwood-inducing techniques is the ordinate, and the agarwood-inducing techniques are the abscissa plot. The results are shown in Figure 1. It can be seen that the content of TT and HL is higher than that of BT and CL, and TT is the highest, BT is the lowest, the content of HL is the most stable, and the content of TT is the most different. The reason is related to the injury type and injury site of the four agarwood-inducing techniques.

### 3.2 Identification and analysis of metabolites in four forms agarwoods

Based on 7 QC samples, peak alignment calibration was performed, and MS-DIAL was used for deconvolution analysis. A total of 147 metabolites were analyzed. Using NIST 17.0 database comparison and the mass spectral retention index (RI), qualitative analysis was used to identify 147 metabolites, and the information is shown in Supplementary Table S2. The identified metabolites mainly include small molecular hydrocarbons, aromatic substances, sesquiterpenoids (C_15_), and a small amount of 2-(2-phenethyl) chromones and diterpenoids.

#### 3.2.1 Small molecule hydrocarbons

**FIGURE 2:**
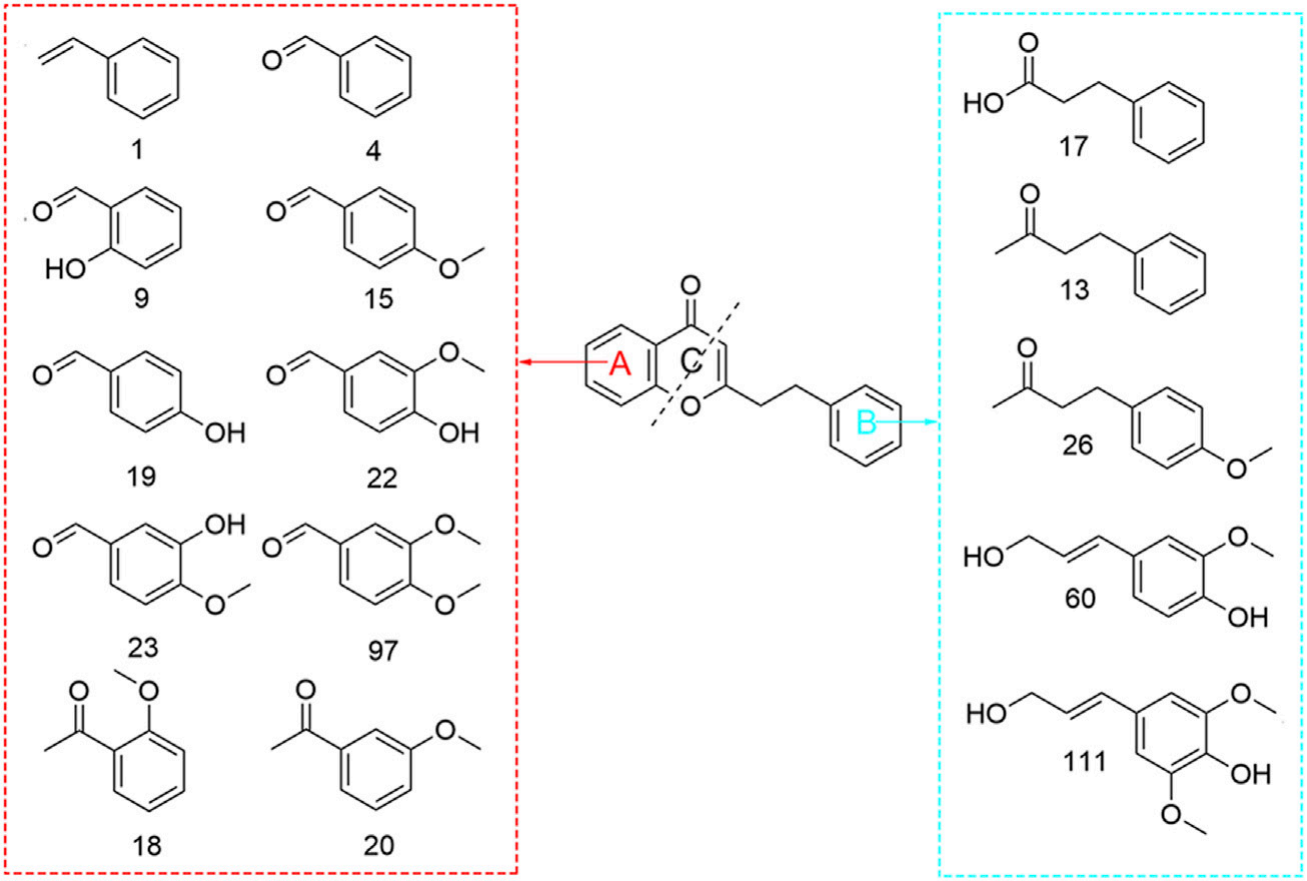
Structural formula of the pyrolysis products of 2-(2-phenylethyl) chromones.

Small-molecule hydrocarbons are ubiquitous in plant volatile metabolites, some of which have a certain effect on the aroma of volatile metabolites, such as aromatic hydrocarbons, because they contain benzene rings, most of which have special aromas (Daba et al., 2022). In this study, a total of 50 hydrocarbons were detected in the volatile metabolites of agarwood produced by four different agarwood-inducing techniques, including 35 aromatic hydrocarbons, 14 ordinary hydrocarbons, and 2 amino (NH_2_)-substituted hydrocarbons. It is worth noting that the structures of 15 substances in aromatic hydrocarbons are similar to those of 2-(2-phenethyl) chromones, a unique substance in agarwood (Figure 2). The possible reason is that 2-(2-phenethyl) chromones are cleaved during extraction (ultrasound) or analysis (injector), which can be proven by Takamatsu’s research. Agarotetrol was heated to 190°C-200°C in this study, and three cleavage products of benzyl acetone **(13)**, benzaldehyde **4)** and benzenepropanoic acid methyl ester were detected from its products (Takamatsu and Ito, 2018).

#### 3.2.2 Sesquiterpenoids

**FIGURE 3:**
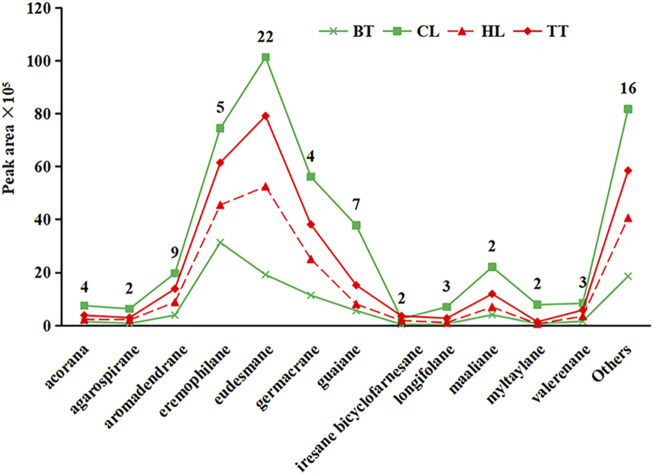
Quantities and contents of different configurations of sesquiterpenoids in four kinds of agarwood

Sesquiterpenoids are the main metabolites of high-boiling volatile metabolites of resin materials and are also the main flavor-adjusting metabolites. Although their skeleton has only 15 carbon atoms, their structures have various configurations. In this study, the configuration of the sesquiterpenoids was confirmed according to the natural product chemistry series “Monoterpenes and Sesquiterpenes Chemistry” edited by Shi Yanping (Shi, 2008; Marangoni et al., 2022). A total of 81 sesquiterpenoids were detected from the 4 forms of agarwood. As shown in Figure 3, 81 sesquiterpenoids can be divided into 28 species of skeletal structure, the number of sesquiterpenes detected in 12 configurations is greater than or equal to 2, and the number of eudesmane-type sesquiterpenes is at most 22, followed by aromadendrane with 9 and 7 of guaiane, 5 of eremophilane, 4 of acorane and germacrane, 3 of longifolene and valerenane, and 2 of the other four configurations, all other 16 skeletal types were detected 1.

As shown in Figure 3, among the volatile sesquiterpenes of agarwood produced by the four agarwood-inducing techniques, except for the contents of eremophilane-type sesquiterpenes contained in BT being smaller than those of eudesmane-type, the eudesmane type was the most abundant type in all samples, followed by the eremophilane type. The contents of other sesquiterpenes were similar among the four agarwood forms. Among the four forms of agarwood, the content of sesquiterpenoids from high to low was CL > TT > HL > BT. The reason for this phenomenon is that CL and TT are inside the tree before harvesting, relatively isolated from the outside environment, and have less natural volatilization and decomposition, but HL and BT are directly exposed to the air, and CL has a longer duration of injury stimulation than TT (Ngadiran et al., 2023), thus, CL has the highest content of sesquiterpenoids, and BT has the lowest content.

#### 3.2.3 2-(2-Phenethyl) chromones

**FIGURE 4:**
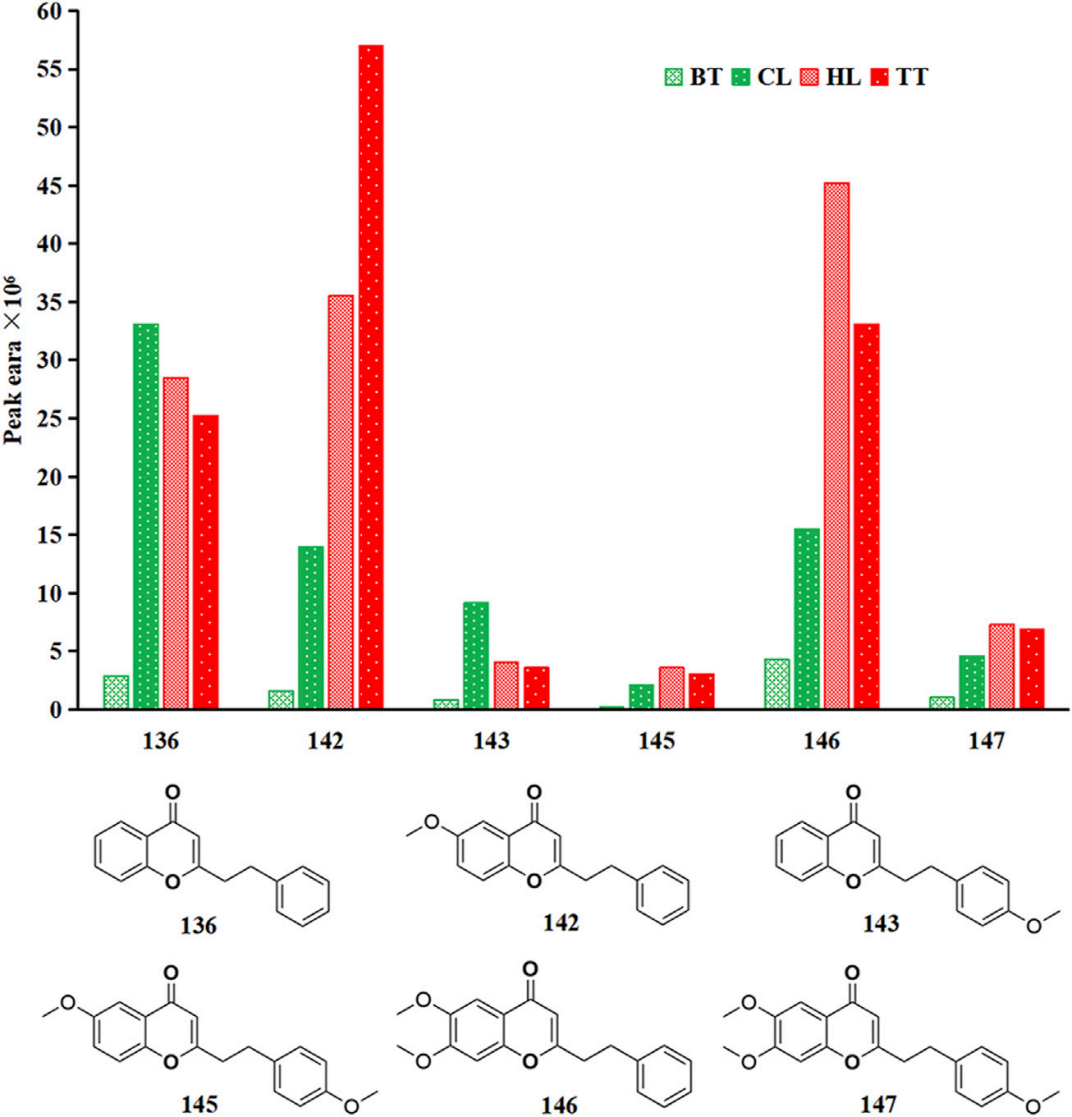
Peak area of 6 kinds of 2-(2-phenylethyl) chromones in four agarwood forms.

2-(2-Phenylethyl) chromone is a class of metabolites formed by the substitution of phenethyl at the 2-position of chromone. It is less distributed in other plants and is mainly found in agarwood. At present, more than 240 kinds of 2-(2-phenylethyl) chromones have been isolated and identified (Pan et al., 2020). Because most of them have one or more hydroxyl, methoxy or chlorine substitutions in the A and B rings, they have a higher boiling point. Therefore, in this study, only 6 2-(2-phenylethyl) chromones were detected from the volatile metabolites of agarwood samples, and they were detected in all four forms of agarwood, but their contents in the four types of agarwood samples were quite different, as shown in Figure 4. Among the four forms of agarwood, 2-(2-phenethyl) chromone, 6-methoxy-(2-phenethyl) chromone and 6,7-dimethoxy-2-(2-phenylethyl) chromone were the main substances, and 6 2-(2-phenethyl) chromones had the highest content in TT, which was 1.04, 1.64 and 11.93 times that of HL, CL and BT, respectively. This is consistent with previous research results. The main reason may be that artificial injury has a stronger degree of injury stimulation in the short term, while natural injury stimulates continuous correction and is milder, and strong injury is more conducive to the activation and expression of polyketide synthase and other 2-(2-phenylethyl) chromone synthases. Artificial induction techniques are more conducive to stimulating the formation of 2-(2-phenylethyl) chromones in *Aquilaria sinensis* (Lour.) Spreng.

### 3.3 Analysis of differential metabolites in agarwood produced by four agarwood inducing methods

#### 3.3.1 Differences in volatile metabolites affected by inducing methods

**FIGURE 5:**
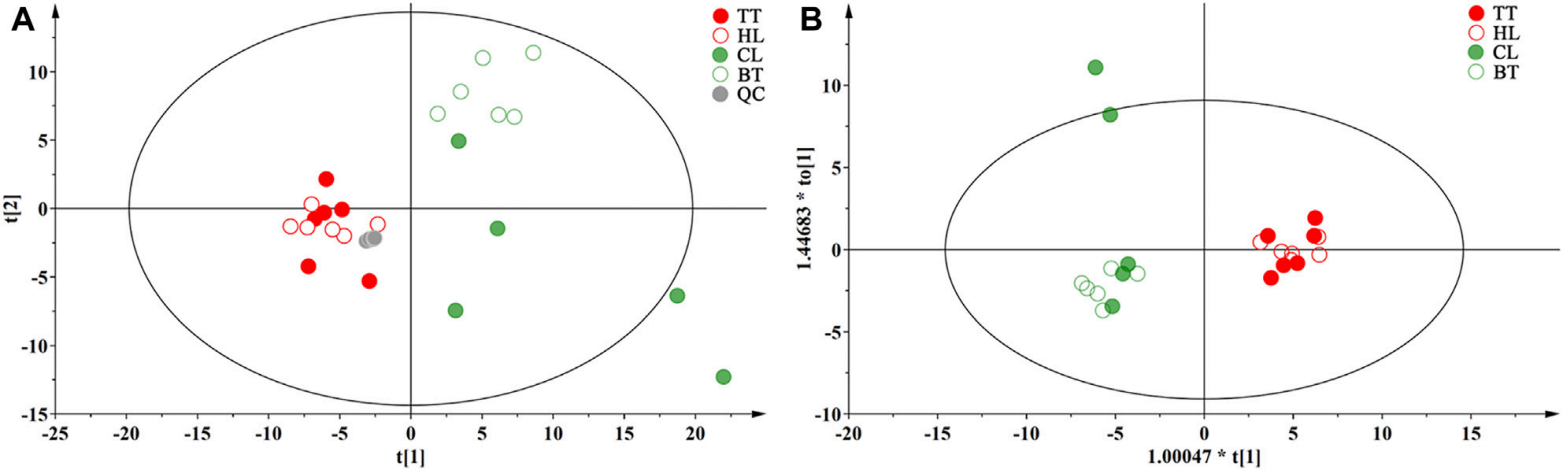
Multivariate statistical analysis results of volatile metabolites of agarwood **(A)**: WIA and AIA PCA loading map, **(B)** WIA and AIA OPLS-DA loading map.

To clarify the influence of the agarwood-inducing technique on the volatile metabolites of agarwood, unsupervised pattern recognition original model of PCA was performed on the 147 metabolites detected by GC‒MS, and the loading diagram was obtained as shown in Figure 5A, they were prescribed by two orthogonal components p (1) and p (2), which accounted for approximately 58.8% of 147 metabolites variances. PCA score showed QC samples are clustered in one point, TT and HL samples are mixed in the left side of the graph, BT is distributed in the upper right side of the graph, and CL is more dispersed, but all are on the right side of the graph. In order to obtain the significant markers of Wild Induced Agarwood (WIA) and Artificial Induced Agarwood (AIA), the supervised OPLS-DA model was further analyzed for the WIA (CL and BT) and AIA (TT and HL) groups, the load diagram is shown in Figure 5B, and model possessed R2 and Q2 values of 0.873 and 0.685, respectively. The model was verified by permutation test to obtain a Q2 value of −0.439, which indicated that the OPLS-DA was good model fitness and predictability. The result showed that the WIA and AIA has a significant distinguish on the volatile metabolites.

#### 3.3.2 Differential metabolite analysis

**FIGURE 6:**
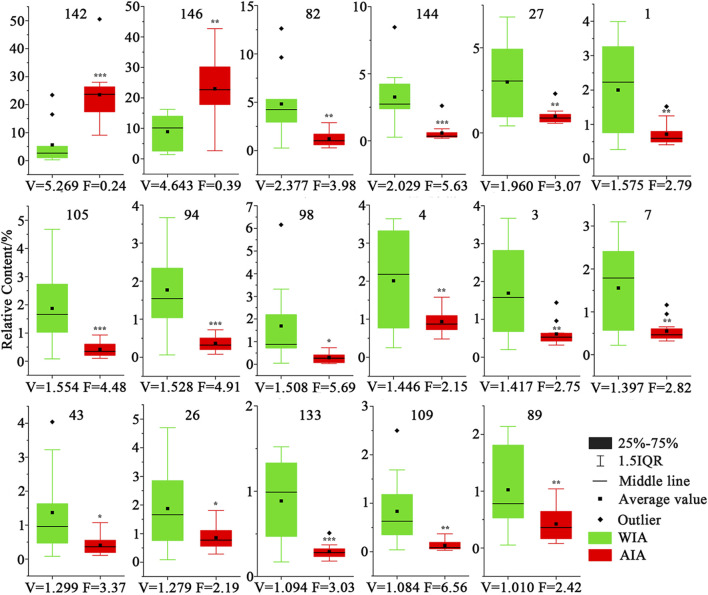
Contents of 17 differential metabolites in AIA and WIA (WIA VS AIA, *mians *p* < 0.05, **mians *p* < 0.01, ***mians *p* < 0.001, V mains VIP, F mians FC).

To further clarify the small difference between the volatile metabolites of agarwood produced by AIA and WIA, on the basis of OPLS-DA, *t*-test *p* < 0.05 and FC > 2 or FC < 0.5, WIA and AIA were screened out. Seventeen main different metabolites were selected, and the relative contents of 17 different metabolites in WIA and AIA are shown in Figure 6. Among them, 6-methoxy-2-(2-phenylethyl) chromone **(142)** and 6,7-dimethoxy-2-(2-phenylethyl) chromone **(146)** were significantly higher in AIA than in WIA, and the relative contents of those two metabolites were higher than 20% in AIA. The other 15 differential metabolites were higher in WIA, but their relative content was relatively stable in AIA, which may be related to the high content of the two metabolites No. **142** and **146** in AIA, indicating that the differences between the two groups of WIA and AIA were mainly produced by 2-(2-phenethyl) chromones.

### 3.4 Network analysis

#### 3.4.1 Construction of volatile metabolites and sleep-promoting, anxiolytic and antidepressant target network

**FIGURE 7:**
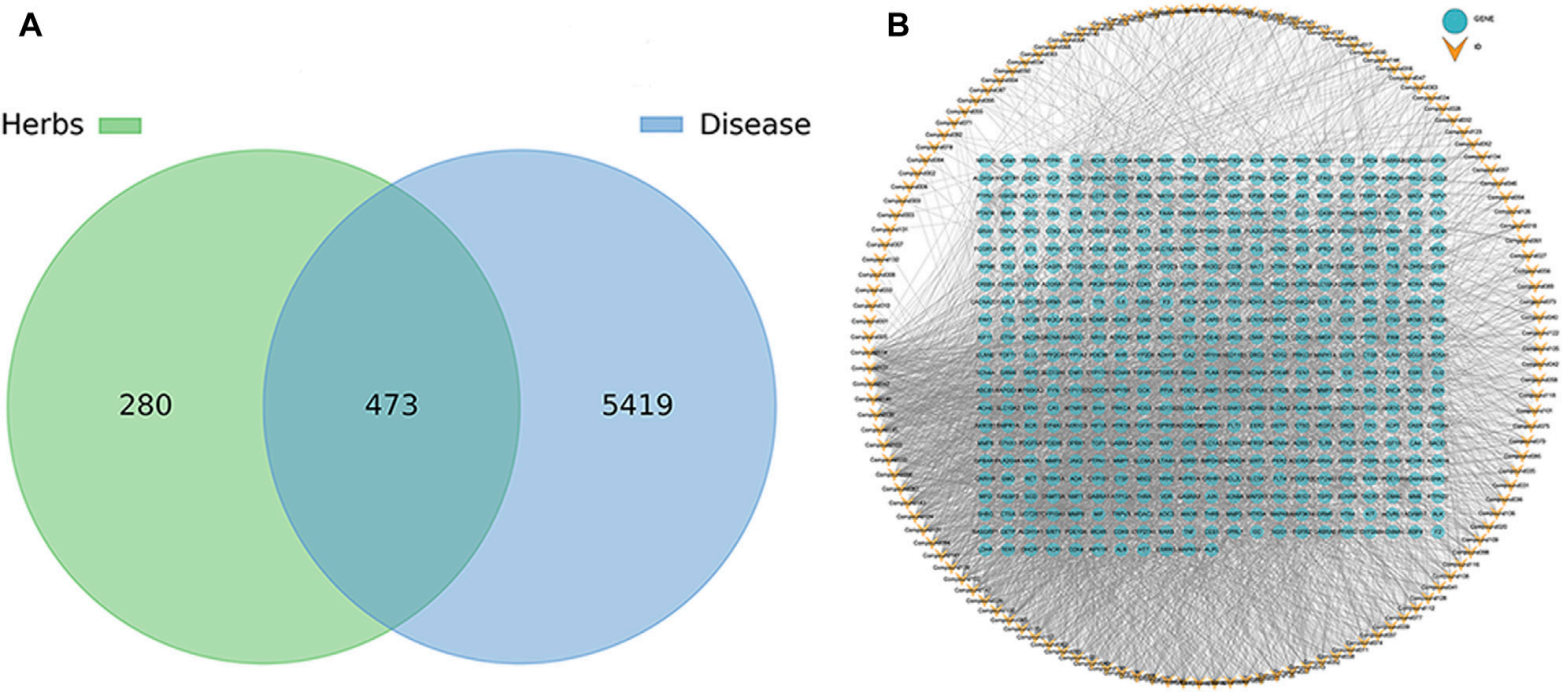
Venn diagram **(A)** and metabolite target network diagram **(B)** of the common targets of pharmacology of volatile metabolites of agarwood and sleep-promoting, anti-anxiety and anti-depressant activities.

A total of 147 metabolites were identified (Supplementary Table S2), which were predicted using Swiss Target Prediction to obtain the target of the metabolite, and the target genes with Probability >0 were selected, and 753 potential targets of the metabolite were predicted (Supplementary Table S2). Among them, the metabolites dodecane **(12)** (E)-4-(3-hydroxyprop-1-en-1-yl)-2-methoxyphenol **(60)**, 2-naphthalenol, 1,2,3,4-tetrahydro-3- (1-hydroxy-1-methylethyl)-5-methoxy **(93)**, trans-sinapyl alcohol **(111)** and anobin **(119)** suggest that no similar active substances have been found, and there is no predictable target gene. Using OMIM and GeneCards database searches for sleep-promoting, anti-anxiety and anti-depression-related disease targets, the search keywords included “Insomnia, Sleep disorders, Sleepiness, Fatigue sedative, Depression, Depressive disorder, Anxiety and Anxiolytic”, and 5892 disease-related targets were obtained. The predicted metabolite potential targets and disease targets were integrated, and a total of 473 shared targets were identified. The results are shown in Figure 7A. The corresponding relationship between metabolites and common targets is further visualized as shown in Figure 7B (Supplementary Figure S1). Twenty-five metabolites have more than 50 targets, and the top five are 1,5-diphenyl-3-pentanone, 1,5-diphenylpent-1-en-3-one, 6-methoxy-2-(2-phenylethyl) chromone, 6,7-dimethoxy-2-(2-phenylethyl) chromone and 2-(2-phenylethyl) chromone.

#### 3.4.2 PPI network construction

**FIGURE 8:**
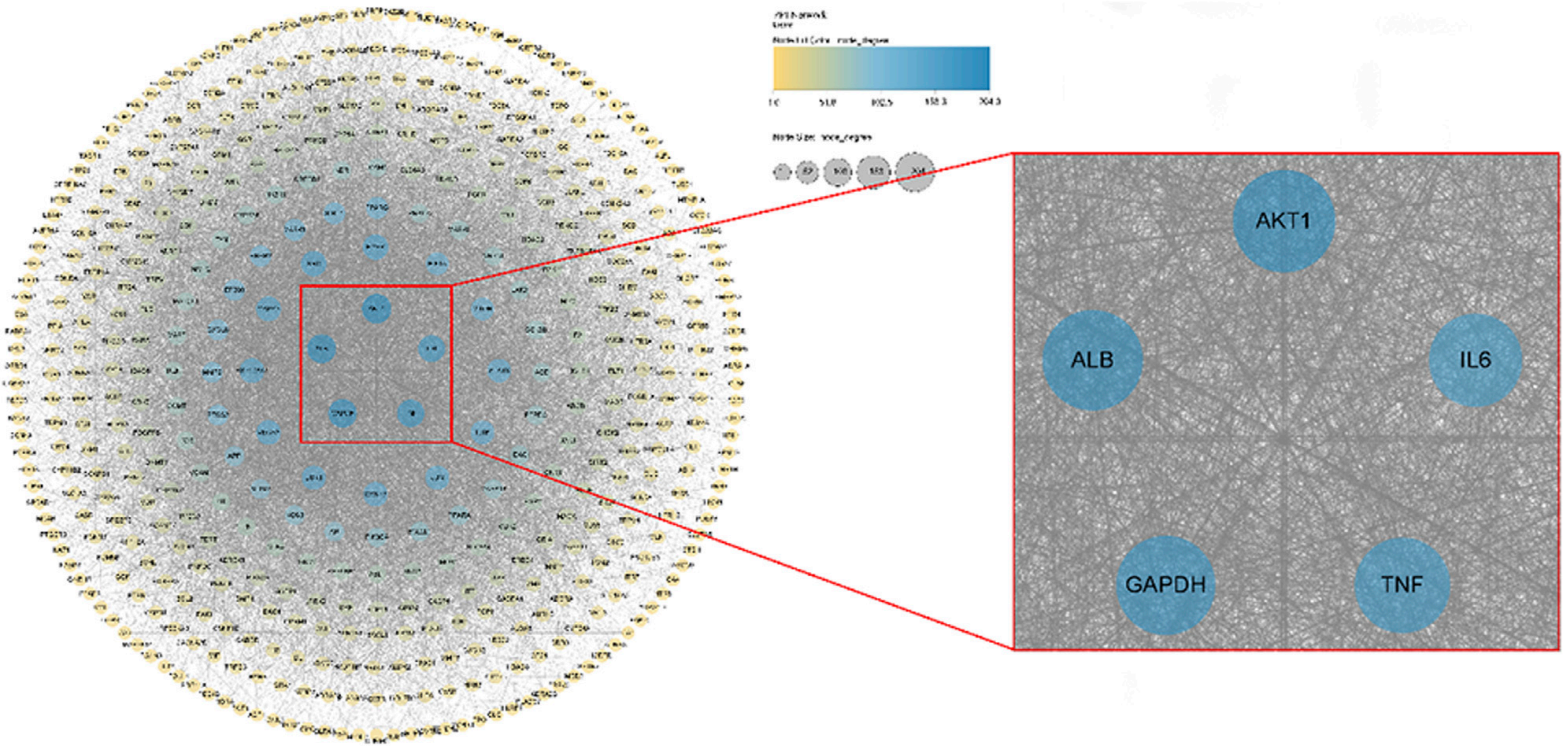
Pharmacology of volatile metabolites of agarwood and potential sleep-promoting, anxiolytic and antidepressant target PPI networks.

The obtained 473 intersection targets were uploaded to the STRING database to construct a protein interaction (PPI) network and imported into Cytoscape 3.9.0 software for visual analysis. The results are shown in Figure 8 (Supplementary Figure S2). According to the node size and color depth, the active proteins mainly affected by the volatile metabolites of agarwood are AKT1, ALB, IL6, GAPDH and TNF.

#### 3.4.3 GO enrichment analysis and KEGG pathway analysis

**FIGURE 9:**
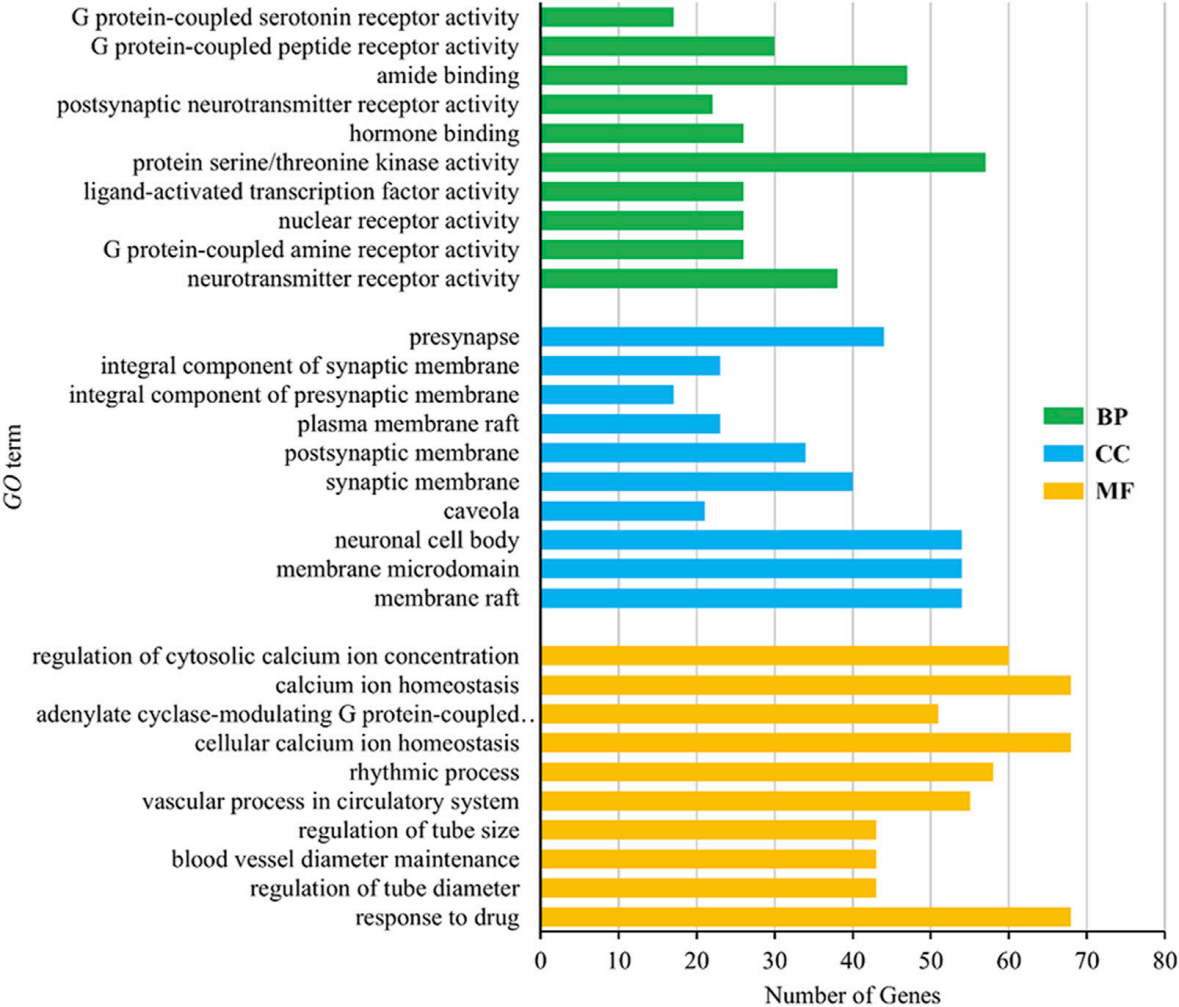
Gene Ontology (GO) enrichment analysis results.

GO analysis included biological process (BP), molecular function (MF) and cellular component (CC). The results showed that there were 2832 items related to BP, mainly including response to drug, regulation of tube diameter, blood vessel diameter maintenance, regulation of tube size and vascular process in the circulatory system, 121 items related to MF, mainly including membrane raft, membrane microdomain, neuronal cell body, caveola and synaptic membrane, 286 items related to CC, mainly including neurotransmitter receptor activity, G protein-coupled amine receptor activity, nuclear receptor activity, ligand-activated transcription factor activity and protein serine/threonine kinase activity, indicating the main activities of the volatile metabolites of agarwood in promoting sleep and anxiolytic/antidepressant activities. The mechanism is to participate in the biological process of the body. In the three methods of BP, MF and CC, the top 10 items were selected according to the *p*-value from small to large for visualization, and the results are shown in Figure 9.

**FIGURE 10:**
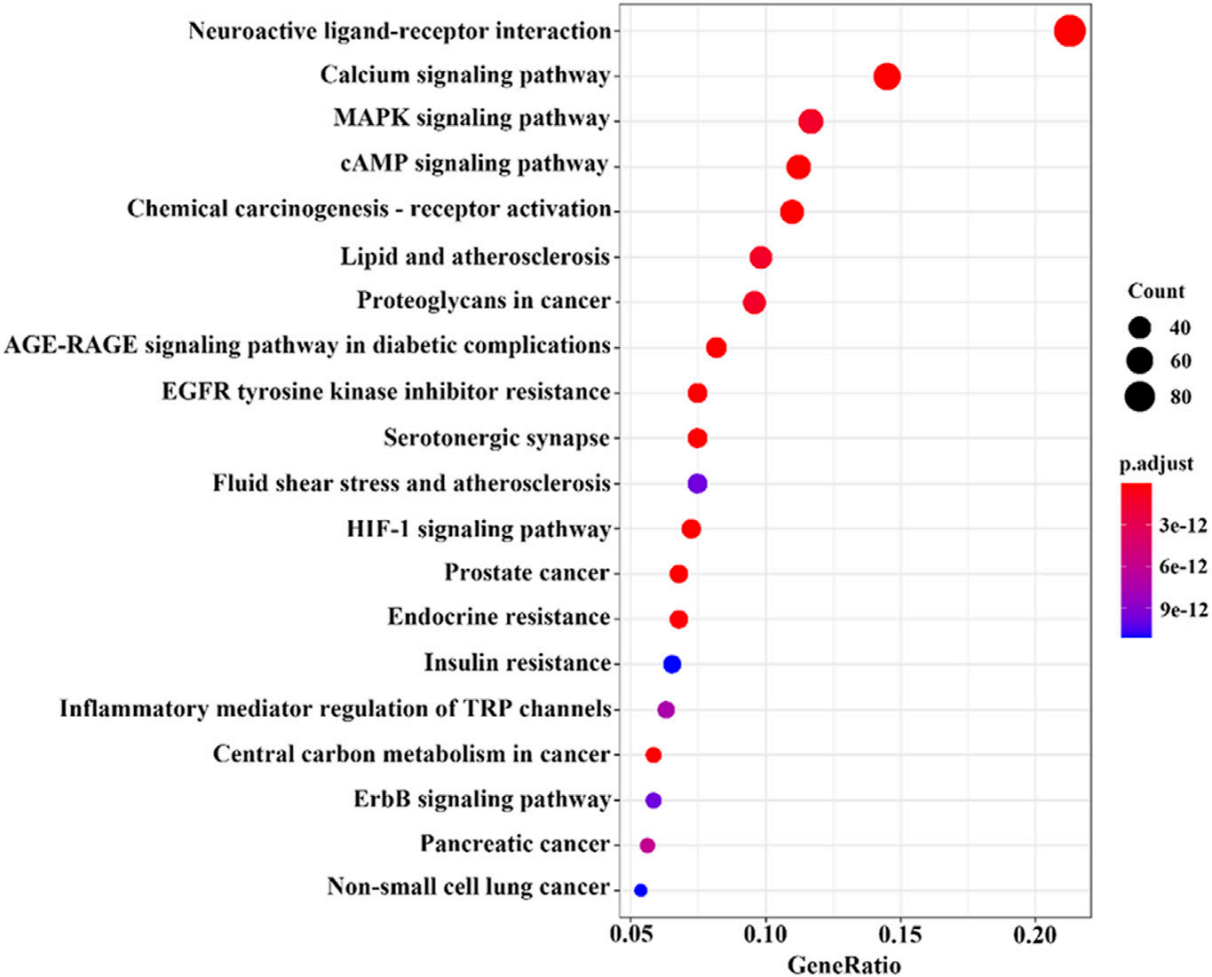
Bubble chart of KEGG analysis results of potential targets.

KEGG analysis showed that 181 cross-targets involved a total of 428 pathways, mainly including neuroactive ligand‒receptor interaction, calcium signaling pathway, EGFR tyrosine kinase inhibitor resistance, AGE-RAGE signaling pathway in diabetic complications, and chemical carcinogenesis-receptor activation (*p*-value <0.05). The count values for the top 20 items and their smaller items are shown in Figure 10. The main effect of the volatile metabolites of agarwood in promoting sleep and anti-anxiety/anti-depressant effects is interactive neural network stimulation.

### 3.5 Molecular docking results

**FIGURE 11:**
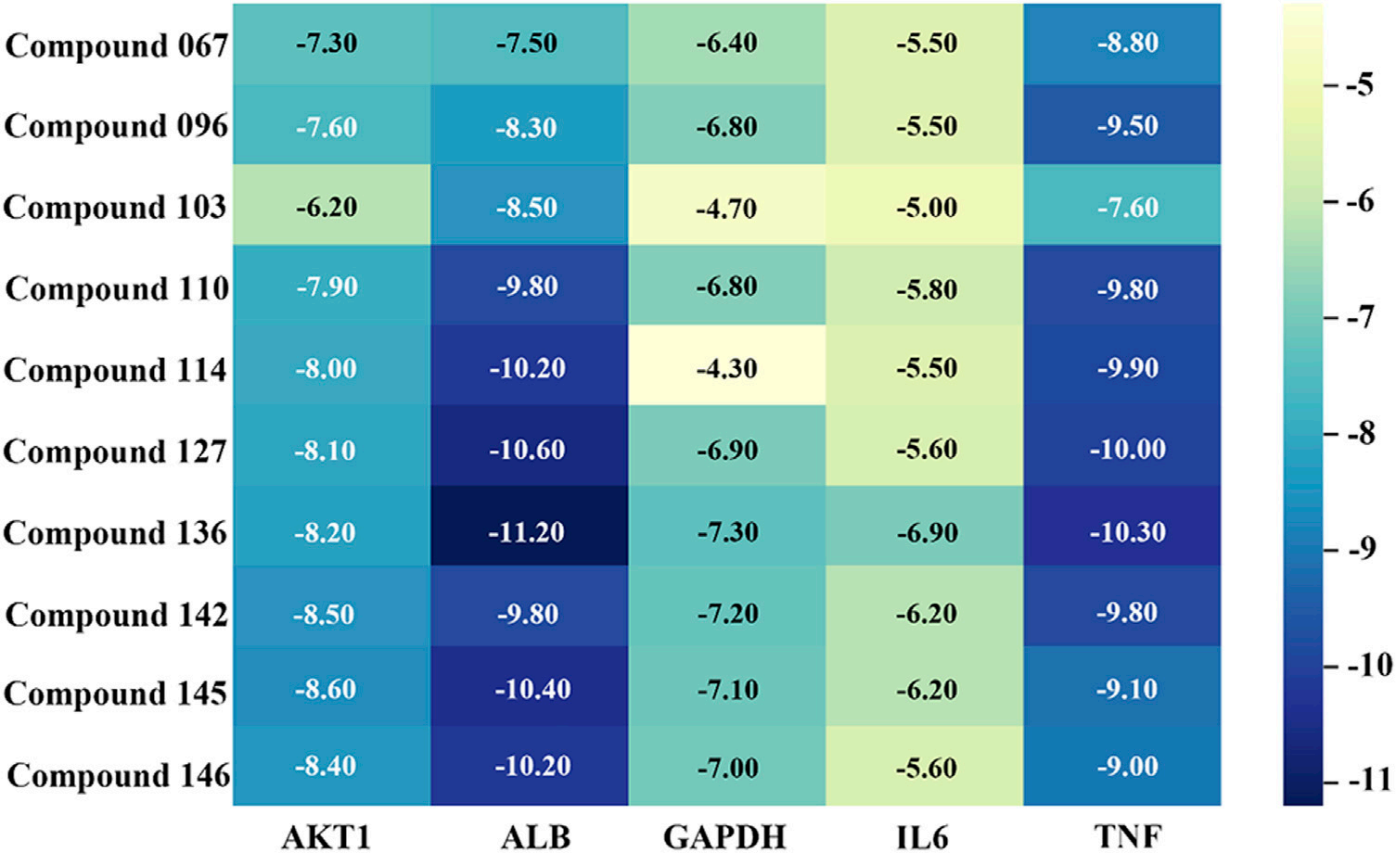
Results of molecular energy binding between key metabolites and target genes.

The top ten metabolites in the compound-target gene regulatory network were selected in descending order of degree value as ligands, and the top five key target genes (AKT1, ALB, GAPDH, IL6, TNF) were selected in descending order of degree value of PPI network analysis results as receptors. The results of molecular docking verification are shown in Figure 11. The set energy of 10 metabolites corresponding to 5 target genes was less than −5 kcal/mol except for metabolites **103** and **114** to GAPDH, indicating that the volatile metabolites of agarwood were well combined with receptor proteins and had high biological activity.

**FIGURE 12:**
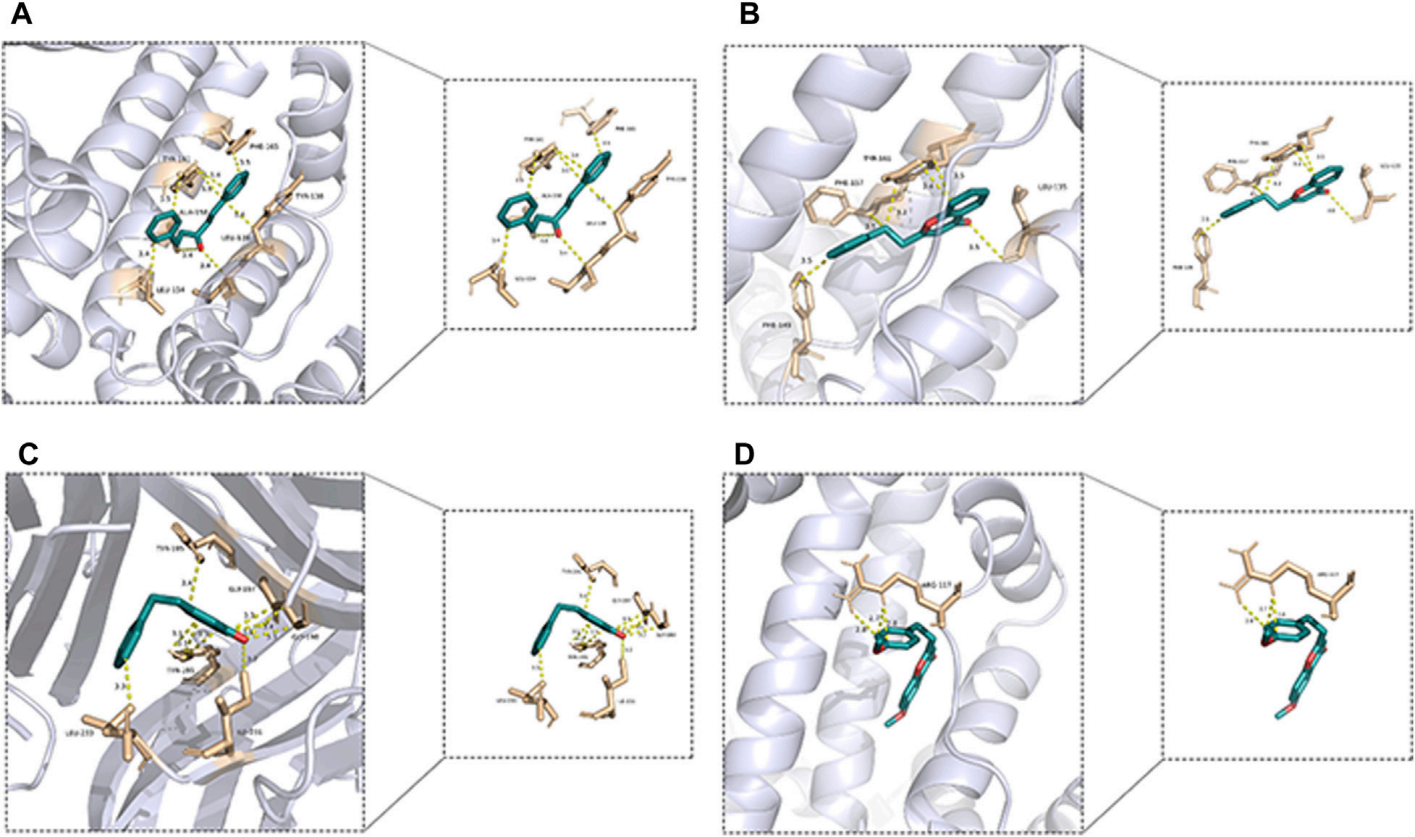
3D molecular docking results. **(A)**: ALB-136, **(B)** ALB-127, **(C)** TNF-136, **(D)** ALB-145)

The top 4 histones and active metabolites with the highest binding activity were selected to visualize the results using PyMOL Version 2.0, as shown in Figure 12. 2-(2-Phenylethyl) chromone **(136)** pairs of ALB have four active sites of PHE-149, PHE-157, TYR-161 and LEU-35, 1-Penten-3-one,1,5-diphenyl **(127)** pairs of ALB have five active sites of PHE-165, TYR-161, TYR-138, ALA-158, LEU-139 and LEU-154, 6-methoxy-2-[2-(4′-methoxyphenethyl) chromone **(145)** pairs of ALB have only one active site of ARG-117, and 2-(2-Phenylethyl) chromone **(136)** pairs of TNF have five active sites of LEU-233, ILE-231, TYR-195, GLY-197 and GLY-198, all of which are linked by one or more hydrogen bonds. It is worth noting that 2-(2-phenylethyl) chromone **(136)** has multiple active sites with both ALB and TNF targets, while 6-methoxy-2-[2-(4′-methoxyphenethyl) chromone **(145)** with only one active site on the ALB target can have a higher binding capacity.

## 4 Discussion

### 4.1 The content of volatile metabolites in agarwood is significantly affected by the induction technique

The content of volatile metabolites in agarwood is a key indicator for judging the quality of agarwood and the harvest period. In this study, TT had the highest content of volatile metabolites, followed by HL and CL, and BT, agarwood produced by artificial techniques is superior to natural agarwood in terms of volatile metabolite content, this is related to the mechanisms of action of the four agarwood-inducing techniques and the positions of the agarwood they produce.

For the artificial inducing agarwood, the “Agar-Wit” technique is based on the drip injection of agarwood-inducing liquid, and through the transpiration of plants, the agarwood-inducing liquid is transported in the plant (Wei et al., 2012; Liu et al., 2013). The damage is mainly concentrated in the inter-xylary phloem of the plants, and the damage area is large (Liu et al., 2019). Therefore, the shape of TT is mainly smooth and slightly curved flakes, and the agarwood produced is relatively isolated from the external environment before it is harvested, thus, the agarwood has the highest content of volatile metabolites. The second highest content of volatile metabolites is the agarwood induced by the “burning-chisel-drilling” technique. Their mechanism is different from that of the “Agar-Wit” technique, and its stimulation and damage are mainly caused by high-temperature roasting and physical damage (Wei et al., 2009; Jim, 2015). Therefore, HL is mainly distributed in the wood phloem, wood rays and conduits around the damage hole, but its damage diffusion ability is uneven (Xiang et al., 2016). Therefore, its shape is mainly blocky, containing cylindrical holes, and the resin around the hole is heavier and gradually decreases outward. The agarwood produced is partially exposed to the external environment, thus, the content of volatile metabolites of HL is slightly lower than that of TT but higher than that of natural agarwood.

For the natural inducing agarwood, CL is induced by secretion and accumulation around the insect tunnel after the boring insect eats the plant and triggers the defense response of the plant (Gu et al., 2017), therefore, the properties of CL are quite different, similar to the shape of wormholes, and contain curved cavities. The volatile metabolite content of CL is quite different depending on insect species and the degree of burrowing. The lowest content of volatile metabolites was found in BT, due to its small degree of damage and the large damaged surface, the shape of BT was mainly in the form of a large uneven plate, and the agarwood it produced was completely exposed to the air, so the volatile content of BT was the lowest.

### 4.2 The volatile composition of four kinds of agarwood, mainly sesquiterpenes

A total of 147 metabolites were detected from the volatile metabolites of four types of agarwood, mainly including small molecule aromatic hydrocarbons, sesquiterpenes, 2-(2-phenylethyl) chromones and a small amount of diterpenes and fatty acids. Among them, 81 kinds of sesquiterpenoids, 55.10% of 147 volatile metabolites, are a main metabolite of agarwood volatile metabolites, which are similar to those reported in existing studies (Tajuddin et al., 2013; Kuo et al., 2019). The number of small-molecule aromatic hydrocarbons is 38, the number of unique 2-(2-phenethyl) chromones in agarwood is 6, and there are 5 enzymatic precursor framework substances for the synthesis of 2-(2-phenylethyl) chromones, which are **114**, **125**, **127**, **128** and **129** (Fan et al., 2022; Xiao et al., 2022), and a few long-chain aliphatic hydrocarbons (Kant and Kumar, 2022).

Eighty-one sesquiterpenes can be divided into 28 skeleton types, and 34 sesquiterpenes in the eucalyptane, guaiacol and erimorphane skeleton types are important metabolites of sesquiterpenes. This result can be proven by reports on phytochemical separation. As of 2021, a total of 182 sesquiterpenoids have been isolated and identified from agarwood, including 134 sesquiterpenes of eucalyptane, guaiacol and erimorphane, accounting for 73.6% (Gao et al., 2019; Li et al., 2021). It is worth noting that there have been no reports on the separation of aramadendrane-type sesquiterpenoids from agarwood, but this has been reported in the GC‒MS analysis of the volatile metabolites and essential oils of agarwood (Kadir et al., 2020; Kao et al., 2021). The reason may be related to the instability of the three-membered ring in the sesquiterpenes of the aramadendrane-type, which is easily fissioned into the guaiane-type in the separation of the metabolites (Moreno-Dorado et al., 2003).

The sesquiterpenes of eudesmane, guaiane and eremophilane are also the main contributors to the aroma of volatile metabolites in agarwood. The eudesmane-type is mainly characterized by fresh, sweet floral, honey, and mint fragrance (Ishihara et al., 1993), the eremophilane-type is mainly characterized by a strong woody aroma, mainly camphor, amber and other strong incense aromas (Näf et al., 1995), and the scent of the guaiane-type is between the eudesmane and eremophilane-types, with a slight woody camphor aroma and a persistent, powerful honey aroma (Wood et al., 2008). At the same time, agarospirane-type sesquiterpenes, which are considered to be the main contributors to the spicy scent of agarwood (Naef, 2010), were also detected 2.

### 4.3 2-(2-phenylethyl) chromones is the main different volatile metabolites in four kinds agarwood

Based on the types and relative contents of the volatile metabolites of agarwood produced by the 4 forms of agarwood, this study used multivariate statistical analysis to analyze the differences. The differences in the composition of the volatile metabolites of the four kinds of agarwood were small, and the volatile metabolites of BT were slightly lower than those of the other three kinds of agarwood. The difference was mainly in the relative content of volatile metabolites. Four 2-(2-Phenethyl) chromones had the highest content in TT, followed by HL, and the content in wild agarwood was significantly lower than that of artificial agarwood, especially 6-methoxy-2-(2-phenylethyl) chromone **(142)** and 6,7-dimethoxy-2-(2-phenylethyl) chromone **(146)**. 2-(2-Phenethyl) chromones are a special class of substances. The current research reports mainly regard agarwood, and only 11 2-(2 phenethyl) chromones have been isolated and detected in other genera of plants. These 11 2-(2 phenethyl) chromones are also found in agarwood (Wang et al., 2000; Yoon et al., 2006; Ibrahim, 2014). 2-(2-Phenethyl) chromones have poor thermal stability, and studies have shown that 2-(2-phenethyl) chromones undergo C-ring cleavage under heating conditions, resulting in benzyl acetone and benzaldehyde-based aromatic small molecules (Hashimoto et al., 1985; Naef, 2010; Takamatsu and Ito, 2018), such as the 15 metabolites detected in this article (Figure 2). Benzyl acetone **(13)** is a substance with floral, herbal and jasmine aromas. Studies have shown that benzyl acetone can strongly inhibit the activity of tyrosinase, and the inhibitory effect is reversible, while tyrosinase is a key enzyme in melanin synthesis (Liu et al., 2015). This is a good explanation for the sleep-promoting and antidepressant effects of 2-(2-phenethyl) chromone substances in agarwood aromatherapy, and benzaldehyde **(4)**, as another major cleavage product, also has cherry and nut aromas and good enzyme inhibitory activity (Loch et al., 2016; Almeida et al., 2022). This is also the main way small molecular aromatic hydrocarbons contribute to the aroma of agarwood. Thus, the main difference in volatile composition and aroma is from 2-(2-phenethyl) chromones and their cracked metabolites.

### 4.4 2-(2-phenylethyl) chromones are the main active substances of agarwood in promoting sleep and anti-anxiety and depression

This study further used network analysis to study the biological activities of the 147 detected volatile metabolites in sleep-promoting, anti-anxiety and anti-depressant activities. Among the 147 substances, the active targets of 142 substances overlapped with the active targets of sleep-promoting, anti-anxiety and anti-depressant activities, reaching 473. The main active proteins affected were AKT 1, ALB, IL6, GAPDH and TNF, especially ALB and TNF, which had the highest activity. ALB is a major protein in the blood circulation (National Library of Medicine, 2024), the metabolites binds well to the ALB protein, indicating its ability to more effectively enter brain tissue through the bloodstream circulation, ALB also has been reported to act on the nervous system and neutralize the free oxygen entering the brain barrier, lowering the oxidative level in the brain environment. (Zhang et al., 2017). TNF and IL-6 is a major inflammatory factor in nerve cells, and is one of the cytokines that make up the acute phase reaction (Hasan et al., 2020), the binding with the pharmacologically active substance can effectively reduce the levels of neurocellular stress, maintaining cellular homeostasis. AKT 1 can greatly affect the level of neuroinflammation in the body, thereby affecting the regulation of the nervous system (Tian et al., 2020). GAPDH is an oxidative stress sensor, an important property of GAPDH is its ability to form aggregates in response to oxidative stress (Nakajima et al., 2017), then triggers apoptosis and mitochondrial dysfunction (Lazarev et al., 2015), it is important for nerve cell integrity homeostasis. Shows that agarwood has good potential in aromatherapy for anti-anxiety and anti-depression.

The top 4 metabolites (**136**, **142**, **145** and **146**) with the lowest binding energy and the best activity potential was 2-(2-phenylethyl) chromones, especially metabolites **142** and **146** are differential metabolites for WIA and AIA, with significant differences in content between the two types of agarwood. This suggests that there are differences in activity between the two types of agarwood in aromatherapy applications, and the main differential substance basis is 2-(2-phenylethyl) chromones substances.

### 4.5 AIA has better sleep-promoting, anti-anxiety and anti-depressant activities than WIA, and TT is the best

At the same time, this study also showed that the main difference in the effects of the four forms of agarwood on the volatile metabolites is the content of 2-(2-phenylethyl) chromones, and the main active substances of antidepressant activity are also 2-(2-phenethyl) chromones. The agarwood-inducing technique has an effect on the efficacy of agarwood in promoting sleep, anti-anxiety and antidepressant activities, and AIA is better than WIA. This result is consistent with the existing reported animal experiments (Wang et al., 2016). In AIA, TT has a higher content of 2-(2-phenylethyl) chromones than HL, so the activity is slightly better than that of HL.

## 5 Conclusions

This study used metabolomic and network analysis methods to study the effect of agarwood-inducing techniques on the volatile metabolites of agarwood and their anti-anxiety, sleep-promoting and anti-depressant activities. The results show that the volatile metabolites of agarwood produced by artificial techniques are higher than those produced by wild techniques, and the average content of volatile metabolites of agarwood produced by internal agarwood-inducing techniques is the highest. That is, the “Agar-Wit” technique is superior to the “burning-chiseling-drilling” method and the natural agarwood-inducing method. The differences in the volatile metabolites of agarwood produced by the four techniques were small, but their relative content was significantly affected by the induction technique, and the main difference was the relative content of four 2-(2-phenylethyl) chromones (TT > HL > CL > BT), which were unique in agarwood.

The main active substances of the volatile metabolites of agarwood regarding sleep-promoting, anti-anxiety and anti-depression effects are mainly 4 2-(2-phenylethyl) chromones, and the main targets are AKT1, ALB, IL6, GAPDH and TNF target proteins, of which ALB and TNF have the best activity, and the content of 4 different chromones in four forms of agarwood is TT > HL > CL > BT. It is shown that the agarwood produced by the artificial induction technique can completely replace the wild induction technique to achieve the expected therapeutic effect when applied to aromatherapy for the treatment of neurological diseases such as insomnia, anxiety and depression, and the “Agar-Wit” technique represented by the internal induction technique is superior to the traditional “burning-chisel-drilling” technique. This research provides an important reference for the promotion and use of artificial induction techniques and the protection of natural agarwood resources.

## Data Availability

The original contributions presented in the study are included in the article/Supplementary Material, further inquiries can be directed to the corresponding authors.

## Abbreviations

AIA, Artificial Induced Agarwood; BT, BanTou Agarwood; CL, ChongLou Agarwood; HL, HuoLao Agarwood; TT, TongTi Agarwood; WIA, Wild Induced Agarwood.
