# Supplementary material for: Utilizing metabolomics and network analysis to explore the effects of artificial production methods on the chemical composition and activity of agarwood
Source: Front Pharmacol. 2024 May 7;15:1357381. doi: 10.3389/fphar.2024.1357381 (PMC11107428; doi:10.3389/fphar.2024.1357381)
Supplement: Supplementary file 2 [file DataSheet1.docx]

**Supplementary Material**


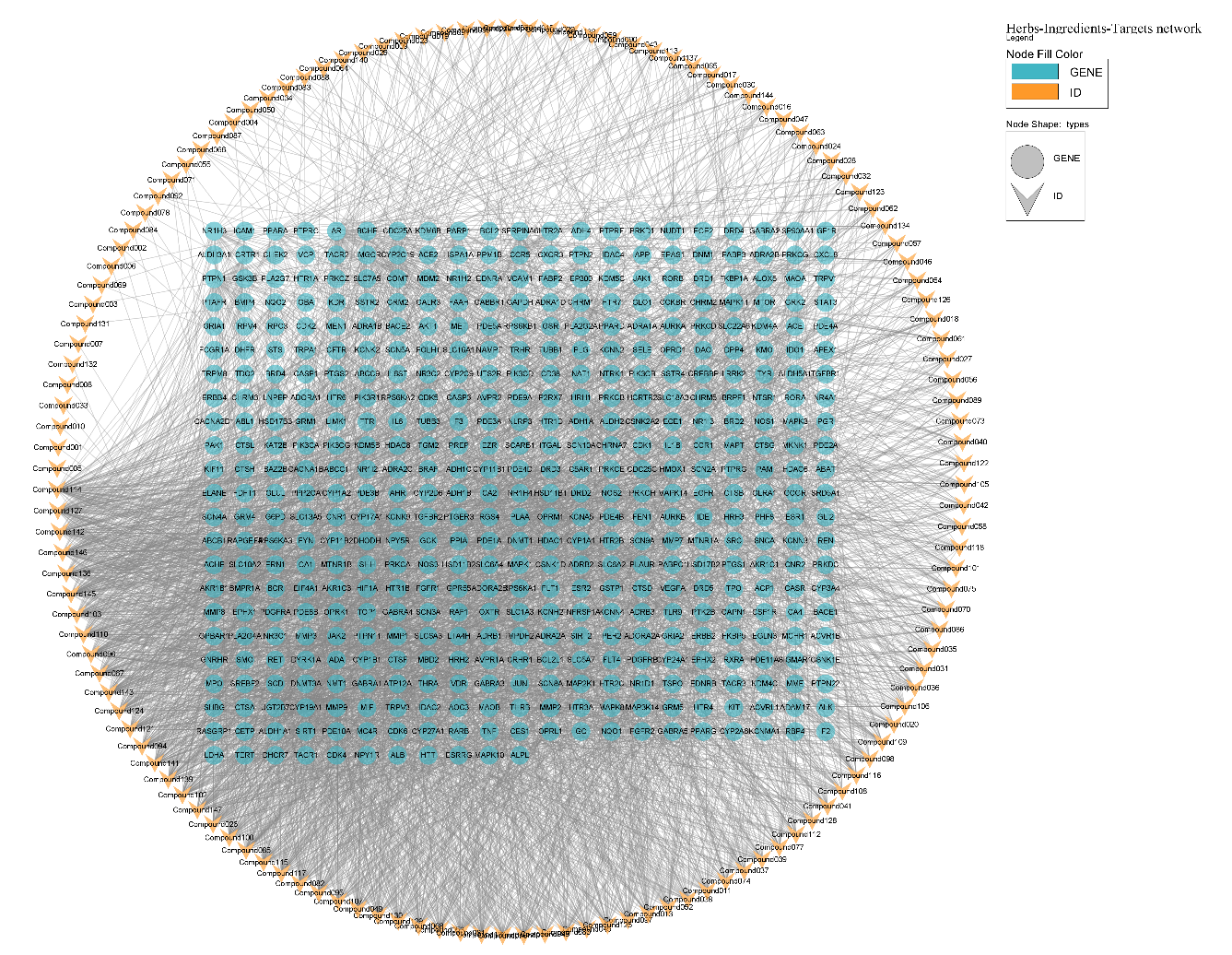


Fig S1 Compound target network diagram (B) of the common targets of pharmacology of volatile components of agarwood and sleep-promoting, anti-anxiety and anti-depressant activities


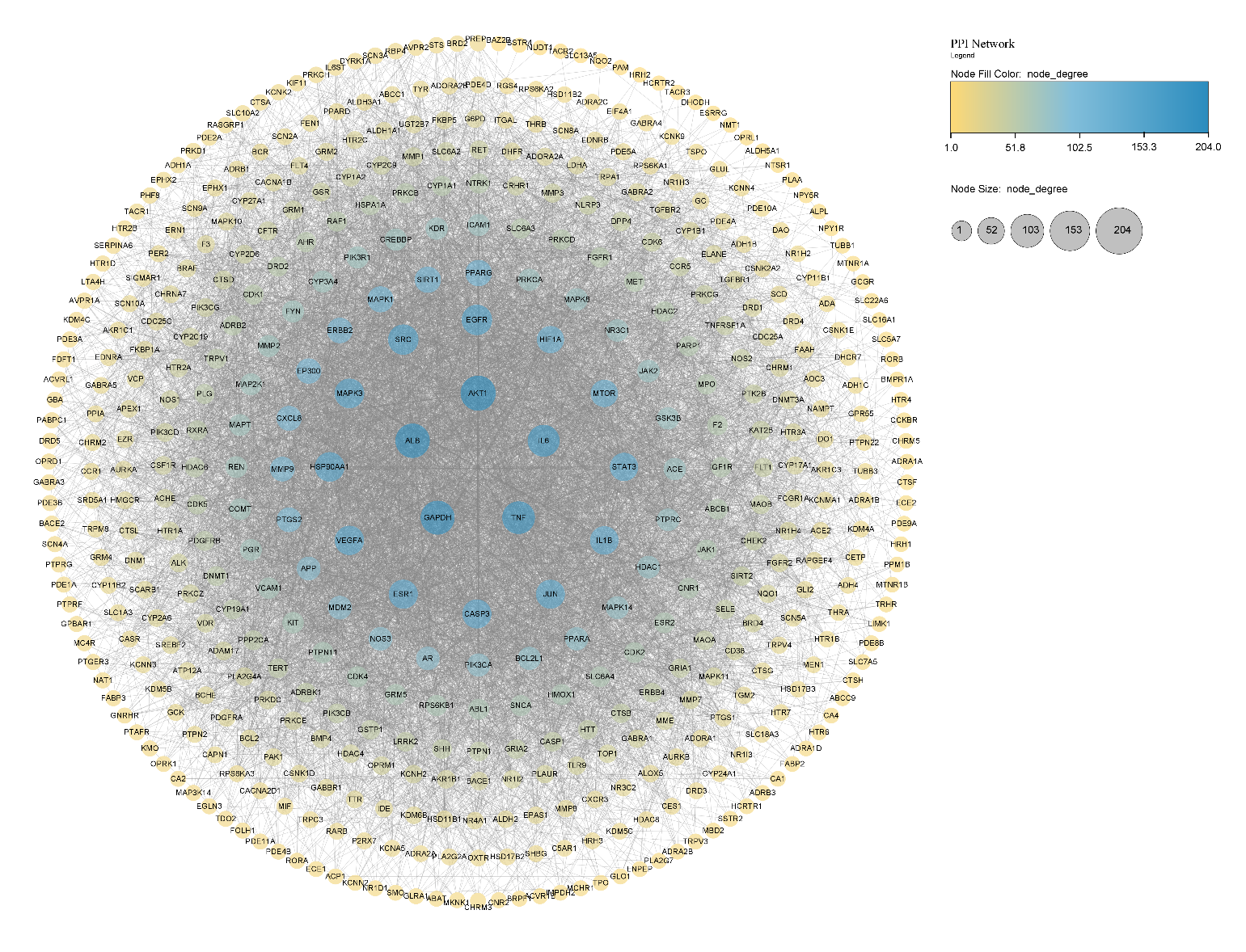


Fig S2 Pharmacology of volatile constituents of agarwood and potential sleep-promoting, anxiolytic and antidepressant target PPI networks
